# Supplementary material for: Causal Relationship between Aging and Anorexia Nervosa: A White-Matter-Microstructure-Mediated Mendelian Randomization Analysis
Source: Biomedicines. 2024 Aug 16;12(8):1874. doi: 10.3390/biomedicines12081874 (PMC11351342; doi:10.3390/biomedicines12081874)
Supplement: Supplementary file 1 [file biomedicines-12-01874-s001.zip › Supplementary Figure/Figure S9/Leaveoneout plot of MR analysis for ubm-b-1924 to AN.pdf]

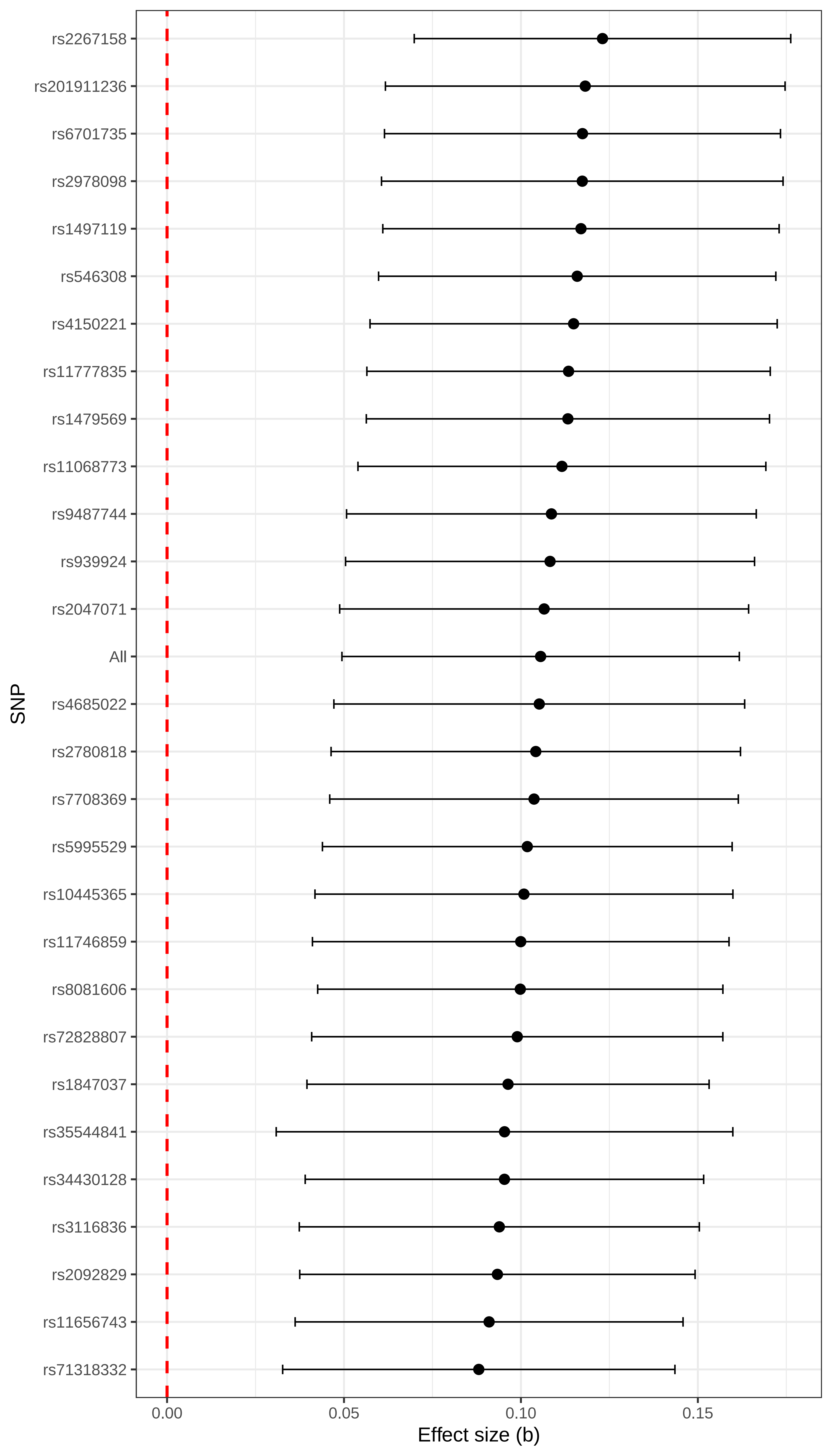

rs2267158
rs201911236
rs6701735
rs2978098
rs1497119
rs546308
rs4150221
rs11777835
rs1479569
rs11068773
rs9487744
rs939924
rs2047071
All
rs4685022
rs2780818
rs7708369
rs5995529
rs10445365
rs11746859
rs8081606
rs72828807
rs1847037
rs35544841
rs34430128
rs3116836
rs2092829
rs11656743
rs71318332
SNP
0.00
0.05
0.10
0.15
Effect size (b)
